# *IL-33* Gene Polymorphisms as Potential Biomarkers of Disease Susceptibility and Response to TNF Inhibitors in Rheumatoid Arthritis, Ankylosing Spondylitis, and Psoriatic Arthritis Patients

**DOI:** 10.3389/fimmu.2021.631603

**Published:** 2021-06-11

**Authors:** Milena Iwaszko, Joanna Wielińska, Jerzy Świerkot, Katarzyna Kolossa, Renata Sokolik, Bartosz Bugaj, Monika Chaszczewska-Markowska, Sławomir Jeka, Katarzyna Bogunia-Kubik

**Affiliations:** ^1^ Laboratory of Clinical Immunogenetics and Pharmacogenetics, Hirszfeld Institute of Immunology and Experimental Therapy, Polish Academy of Sciences, Wrocław, Poland; ^2^ Department of Rheumatology and Internal Medicine, Wroclaw Medical University, Wrocław, Poland; ^3^ Department of Rheumatology and Connective Tissue Diseases, Jan Biziel University Hospital No. 2, Bydgoszcz, Poland; ^4^ Ludwik Rydygier Collegium Medicum in Bydgoszcz, Nicolaus Copernicus University, Toruń, Poland

**Keywords:** rheumatoid arthritis, psoriatic arthritis, ankylosing spondylitis, spondyloarthritis, *IL-33* gene polymorphism, anti-TNF therapy, TNF inhibitors

## Abstract

**Objective:**

Rheumatoid arthritis (RA), ankylosing spondylitis (AS), and psoriatic arthritis (PsA) belong to inflammatory rheumatic diseases, the group of conditions of unknown etiology. However, a strong genetic component in their pathogenesis has been well established. A dysregulation of cytokine networks plays an important role in the development of inflammatory arthritis. Interleukin 33 (IL-33) is a recently identified member of the IL-1 family. To date, the significance of IL-33 in inflammatory arthritis has been poorly studied. This research aimed to investigate the potential of *IL-33* gene polymorphisms to serve as biomarkers for disease susceptibility and TNF inhibitor response in RA, AS, and PsA patients.

**Materials and Methods:**

In total, 735 patients diagnosed with RA, AS, and PsA and 229 healthy individuals were enrolled in the study. Genotyping for three single nucleotide polymorphisms (SNPs) within the *IL-33* gene, namely, rs16924159 (A/G), rs10975519 (T/C), and rs7044343 (C/T), was performed using polymerase chain reaction amplification employing LightSNiP assays.

**Results:**

In the present study, the *IL-33* rs10975519 CC genotype was associated with a decreased risk of developing RA in females, while the *IL-33* rs16924159 polymorphism was associated with the efficacy of anti-TNF therapy and clinical parameters for RA and AS patients. The *IL-33* rs16924159 AA genotype correlated with higher disease activity and worse clinical outcomes in RA patients treated with TNF inhibitors, and AS patients carrying the *IL-33* rs16924159 AA genotype had higher disease activity and a worse response to anti-TNF therapy. That indicates a deleterious role of the *IL-33* rs16924159 AA genotype in the context of RA, as well as AS.

**Conclusions:**

The obtained results suggest that *IL-33* gene polymorphisms might be potential candidate biomarkers of disease susceptibility and anti-TNF treatment response in patients with inflammatory rheumatic diseases.

## Introduction

Inflammatory arthritis comprises a diverse group of rheumatic diseases characterized by inflammation of synovial joints and systemic manifestations. Rheumatoid arthritis (RA), psoriatic arthritis (PsA), and ankylosing spondylitis (AS) constitute the most common subtypes of inflammatory arthritis. The worldwide prevalence of inflammatory arthritis amounts to approximately 3%. The exact etiology of RA, PsA, and AS has not been elucidated; however, there is evidence that genetic factors may contribute to their development.

An imbalanced cytokine network plays a crucial role in the pathogenesis of inflammatory arthritis (1, 2). Interleukin-33 (IL-33) constitutes a recently identified member of the IL-1 family that includes IL-1α, IL-1β, IL-1Ra, and IL-18. IL-33 is a dual-function protein, acting both as an endogenous danger signal and a nuclear factor (3, 4). This cytokine is constitutively expressed in the nuclei of endothelial and epithelial cells, acts as a nuclear repressor factor, and is involved in gene transcription regulation (5–7). In response to cellular damage, full-length IL-33 is rapidly released into the extracellular matrix, initiating an inflammatory response (8, 9). This cytokine functions as an alarmin, alerting the immune system and triggering the inflammatory process (8, 10).

IL-33 is predominantly expressed in epithelial, endothelial, and immune cells, including dendritic cells, macrophages, mast cells, and activated Th2 cells. IL-33 interacts with a receptor complex formed by orphan receptor ST2 (also known as IL-1RL1) and IL-1 receptor (IL-1RAcP) (11, 12). Two major ST2 isoforms result from the alternative splicing of gene transcripts: a full-length transmembrane form (ST2L) and a soluble form (sST2) (9). sST2 does not possess transmembrane and cytoplasmic domains and functions as a decoy receptor. IL-33 binding by sST2 inhibits the IL-33/ST2L interaction and, subsequently, IL-33–mediated signaling (13). ST2L expression has been detected for various types of immune cells: natural killer (NK) and NKT cells, innate lymphoid cell type II (ILC2), Th2 lymphocytes, dendritic cells, macrophages, regulatory T cells (Treg), B cells, eosinophils, basophils, and mast cells.

Following IL-33/ST2L binding, co-receptor recruitment of IL-1R accessory protein (IL-1RAP) is indispensable for signal transduction (14). The IL-33/ST2/IL-1RAcP complex induces recruitment of an adaptor protein, myeloid differentiation factor 88 (MyD88), that results in the activation of interleukin-1 receptor-associated kinases 1 and 4 (IRAK1/4) and tumor necrosis factor (TNF) receptor-associated factor 6 (TRAF6). These proteins trigger a downstream activation of the nuclear factor-κB (NF-κB) and the mitogen-activated protein kinase (MAPK) pathways (involving p38, JNKs c-Jun N-terminal kinases, and ERK extracellular signal-regulated kinase), leading to a proliferation of pro-inflammatory cytokine synthesis (9, 15).

IL-33 is predominantly involved in the Th2-mediated immune response by inducing IL-4, IL-5, and IL-13 cytokine production (16) and is crucial for the activation of Th2 cells, mast cells, eosinophils, basophils, dendritic cells (DC), and ILC2 cells (17). IL-33 was initially regarded as a mediator of the Th2-mediated immune response; however, it has also been implicated in Th1-associated immunity (3, 18). IL-33–mediated signaling is involved in the activation of Th1 cells, CD8+ T cells, NK and NKT cells, neutrophils, macrophages, and B cells (19–23). Moreover, IL-33 enhances the Th17 immune response, promotes an expansion of regulatory T (Treg) cells and DCs differentiation (24–27). The IL-33/ST2L axis is also involved in the generation of cytokines with pro-inflammatory potential, including IL−1β, IL-6, and tumor necrosis factor-α (TNF-α) (24, 28, 29).

IL-33 constitutes an essential component of both innate and adaptive immunity (6). This pleiotropic cytokine can target a broad range of immune cells and exerts multiple effects on immune system functions. The pleiotropic character of IL-33 indicates its potential as an essential player in the pathogenesis of autoimmune diseases. It has been revealed that dysregulation within the IL-33/ST2L axis contributes to the development of various diseases, including cardiovascular disorders, cancer, and infectious diseases (9, 15, 19). An increasing body of evidence also implies a crucial role of IL-33 in the pathogenesis of autoimmune disorders, such as inflammatory bowel disease, multiple sclerosis, psoriasis, and diabetes (9, 15).

This research aimed to investigate the potential of *IL-33* gene polymorphisms as predictors of disease susceptibility and biomarkers of anti-TNF therapy response in major autoimmune rheumatic diseases, including RA, AS, and PsA.

## Materials and Methods

### Study Group

In total, 964 cases and controls were enrolled, including 466 RA, 143 AS, and 126 PsA patients. The control group consisted of 229 healthy individuals. The patients were recruited from the Department of Rheumatology and Internal Medicine at Wrocław Medical University, Poland, and the Department of Rheumatology and Connective Tissue Diseases at Collegium Medicum, Bydgoszcz, Poland. RA was diagnosed according to the 2010 American College of Rheumatology/European League Against Rheumatism (ACR/EULAR) Classification Criteria for Rheumatoid Arthritis; all patients diagnosed with AS fulfilled the 1984 modified New York Criteria; PsA diagnoses were established according to the Classification Criteria for Psoriatic Arthritis (CASPAR).

The following demographic and clinical data were collected from all studied patients: gender, body mass index (BMI), disease onset, disease duration, medications received, C-reactive protein (CRP) levels, erythrocyte sedimentation rate (ESR), tender joint count (TJC), swollen joint count (STC), rheumatoid factor (RF) levels, anti-cyclic citrullinated peptide antibody (anti-CCP) levels (for RA patients), HLA-B27 status (for AS patients), Disease Activity Score-28 (DAS28), the Bath Ankylosing Spondylitis Disease Activity Index (BASDAI) (for AS and PsA patients), and the visual analog scale (VAS, ranging from 0 to 100 mm) of pain, and health assessment questionnaires (HAQs) and global health assessments (self-reported and from a physician) were used. The detailed demographic and clinical characteristics of the RA, AS, and PsA patients are depicted in **Table 1**.

**Table 1:** Patients’ and controls’ characteristics.

	RA patients N = 466	AS patients N = 143	PsA patients N = 126	Controls N = 229
Demographics				
Sex [females/males (% of females)]	368/98 (79.0)	36/107 (25.2)	62/64 (49.2)	98/131 (42.8)
Age (years) [mean (± SD)]	51.6 (± 12.3)	44.4 (± 13.0)	47.9 (± 12.1)	45.5 (± 11.9)
Clinical data				
Disease duration (years) [mean (± SD)]	12.7 (± 8.1)	12.0 (± 9.8)	10.7 (± 10.3)	
Disease onset (years) [mean (± SD)]	39.1 (± 12.6)	31.9 (± 9.6)	38.3 (± 12.6)	
DAS28 at baseline [mean (± SD)]	6.4 (± 0.7)		5.5 (± 1.3)	
CRP at baseline [mean (± SD)]	23.4 (± 34.0)	30.0 (± 56.7)	12.6 (± 17.3)	
BASDAI at baseline [mean (± SD)]		7.5 (± 1.4)	7.7 (± 1.0)	
VAS at baseline [mean (± SD)]	77.6 (± 11.9)	80.5 (± 10.8)	72.2 (± 16.0)	
HLA-B27 positive [%]		87.2		
RF positive [%]	68.3			
Anti-CCP positive [%]	90.0			
Anti-TNF drugs				
Etanercept [%]	26.6	30.8	32.5	
Adalimumab [%]	18.9	43.4	33.3	
Infliximab [%]	1.7	1.4	1.6	
Certolizumab pegol [%]	3.2	11.9	1.6	
Golimumab [%]		8.4	10.3	
Concomitant treatment				
Glucocorticosteroids [%]	91.0	20.5	21.4	
Methotrexate [%]	92.0	31.1	61.1	

RA, rheumatoid arthritis; AS, ankylosing spondylitis; PsA, psoriatic arthritis; DAS28, disease activity score 28; CRP, C-reactive protein; BASDAI, Bath Ankylosing Spondylitis Disease Activity Index; VAS, visual analog scale; HLA-B27, human leukocyte antigen B27; RF, rheumatoid factor; anti-CCP, anti cyclic citrullinated peptide antibodies; SD, standard deviation.

The eligibility criteria were: age over 18 years; Caucasian ethnicity; confirmed diagnosis of RA, AS, or PsA; resistance to treatment with at least two disease-modifying anti-rheumatic drugs (DMARDs) for RA and PsA patients; resistance to treatment with at least two non-steroidal anti-rheumatic drugs for AS and PsA patients; the presence of active disease before the initiation of anti-TNF therapy; and the commencement of treatment with one of the four anti-TNF biological agents (adalimumab, etanercept, infliximab, certolizumab, or golimumab) at the time of the study. A complete medical history and a physical examination were also required.

The exclusion criteria adopted in the research were as follows: age below 18 years, the coexistence of other autoimmune disorders, infections with hepatotropic viruses or human immunodeficiency virus, infections resistant to therapy, a history of malignancy, the coexistence of other severe acute or chronic medical condition, pregnancy or breastfeeding, alcohol or drug abuse, poor clinical records, and an unwillingness or inability to cooperate.

Patients were examined before commencing the TNF blocking therapy and after 12 and 24 weeks of the treatment. Age- and sex-matched controls (without any family history of rheumatic diseases) from the Blood Bank of Wroclaw served as the control group. All participants were of Caucasian ancestry, and written informed consent was obtained. The study protocol was approved by the Wroclaw Medical University Ethics Committee.

### Anti-TNF Treatment Regimen

The patients were administered the following anti-TNF agents: infliximab, adalimumab, etanercept, and certolizumab pegol. The patients received recommended doses of TNF blockers: 3 mg/kg body weight of infliximab given as intravenous infusions at weeks 0, 2, and 6 and every 8 weeks thereafter, subcutaneous injections of 40 mg adalimumab every other week, 50 mg etanercept every week, and 400 mg certolizumab pegol at weeks 0, 2, and 4 and 200 mg every 2 weeks thereafter, and 50 mg of golimumab once a month. Stable doses of methotrexate (MTX), glucocorticoids, and non-steroidal anti-inflammatory drugs were allowed. Therapeutic responses were assessed at weeks 12 and 24 after anti-TNF therapy initiation.

### Assessment of the Disease Activity and Therapeutic Response

Disease activity in the RA patients was calculated with DAS28. DAS28 is a composite index incorporating four variables: Number of swollen and tender joints, CRP level, and a self-reported global health assessment (VAS, mm). High disease activity was defined by DAS28 > 5.1, moderate by 3.2 < DAS28 ≤ 5.1, and DAS28 ≤ 3.2 referred to low disease activity. EULAR response criteria were used to measure anti-TNF treatment efficacy, which combines the improvement in DAS28 scores between the initial and final score with the DAS28 score at the time of evaluation (30).

Bath Ankylosing Spondylitis Disease Activity Index (BASDAI) was employed to assess AS patient disease activity. BASDAI is a composite index comprising an evaluation using a VAS scale (range, 0–10 cm, where 0 = none and 100 = very severe) of the following components: Fatigue, spinal pain, enthesitis, peripheral joint pain/swelling, morning stiffness duration, and morning stiffness severity. A BASDAI of 50 (at least 50% improvement from baseline BASDAI or absolute score change of 2; scale 0–10) indicates a good response to therapy (31).

In the PsA cohort, DAS28 and BASDAI were used to assess disease activity status. As the anti-TNF therapy outcome measure, the psoriatic arthritis response criteria (PsARC) were employed. These criteria included TJC, SJC (assessing 68 and 66 joints, respectively, including the distal interphalangeal joints in the hands and feet), patient (PtGA) and provider (PrGA) global assessment of disease activity. An improvement of at least 30% in at least one joint count and one other measure and no decrease in the other measurements was defined as a positive therapeutic outcome (32, 33).

### SNP Selection and Genotyping

The selection of genetic variants within the *IL-33* gene was based on an analysis of the available literature and search results from the HapMap and NCBI dbSNP databases. Information of the predicted functional consequences of SNPs was obtained using the SNPinfo Web Server (34). The studied SNPs were characterized with minor allele frequencies above 10% (1000 Genomes Project) (35).

Peripheral venous blood from each subject was collected in ethylenediaminetetraacetic acid (EDTA) anticoagulant tubes. DNA isolation was carried out using a Maxwell 16 Blood DNA Purification Kit (Promega Corp., Madison, WI, USA). The *IL-33* SNPs were detected by real-time PCR using LightCycler Technology with SimpleProbe probes (LightSNiP assays) designed by TIB MolBiol (Berlin, Germany). Genotyping was performed on a LightCycler 480 Real-Time PCR system (Roche Diagnostics, Rotkreuz, Switzerland) according to the manufacturer’s instructions.

### Statistical Analysis

The Population Genetics R package (cran:genetics, version 1.3.8.1) was used for testing the Hardy–Weinberg equilibrium (HWE) of the genotype distributions of the examined *IL-33* polymorphisms. For the presentation of categorical variables, frequencies and percentages were used, while means and standard deviations were calculated for continuous variables. A Fisher’s exact test was used to detect differences in the genotype and allele frequencies between patients and controls. Fisher’s exact or Wilcoxon’s tests were applied to assess the relationships between the *IL−33* genetic variants and clinical parameters. RA patients’ relative changes of DAS28 scores between baseline and weeks 12 as well as 24 were calculated for each patient by dividing a latter value by a baseline one. Analyses of the distributions of the *IL-33* genotypes and alleles within patient groups for their associations with therapeutic effect were performed using Fisher’s exact test. RA patients with a good and moderate EULAR response were grouped together and compared to patients with no response. Stratification analyses employed the Cochran–Mantel–Haenszel χ^2^ test for count data, followed by a post-hoc groupwise association test of genotypes (cran:rcompanion, version 2.3.26). Bonferroni corrected *p*_c_ values were calculated by multiplying the observed *p* value by 27 (3 diseases × 3 outcomes × 3 models). Level of statistical significance was set at 5 % (*p*_c_ < 0.05), uncorrected *p* < 0.05 was considered suggestive. Assuming the aforementioned significance level, the additive model, the minor allele frequency of 0.2, and effect size of 1.50, our study had 55 % power in the AS patients, 53% in the PsA patients, and 82 % in case of the RA patients. All statistical calculations, except power, were performed using R software environment (version 3.6.3; x86_64-pc-linux-gnu) (36). Statistical power calculations were performed with the Genetic Association Study Power Calculator (https://csg.sph.umich.edu/abecasis/cats/gas_power_calculator).

## Results

### Genotype and Allele Distributions of IL-33 Polymorphisms in the RA, AS, PsA Patients and Healthy Controls

The genotype distributions of the examined *IL-33* polymorphisms in control group were in Hardy–Weinberg equilibrium (*p* = 0.88 for rs10975519, *p* = 0.57 for rs16924159, and *p* = 0.67 for rs7044343). The frequency of the genotypes and alleles of the *IL-33* rs10975519, rs16924159, or rs7044343 did not differ between the RA patients and controls, and no significant differences were found between the controls and AS or PsA patients (**Table 2**).

**Table 2:** Gender-stratified analysis of the *IL-33* genotypes frequencies in rheumatoid arthritis, ankylosing spondylitis, and psoriatic arthritis patients.

		rs10975519				rs16924159			rs7044343	
	CC	CT		TT	AA	AG	GG	CC	CT		TT
RA											
Female patients	142 (38.7%)	188 (51.2%)		37 (10.1%)	52 (14.2%)	182 (49.6%)	133 (36.2%)	48 (33.2%)	197 (53.7%)		122 (33.2%)
Male patients	43 (43.9%)	51 (52.0%)		4 (4.1%)	13 (13.3%)	46 (46.9%)	39 (39.8%)	7 (7.1%)	55 (56.1%)		36 (36.7%)
Female controls	53 (54.1%)	34 (34.7%)		11 (11.2%)	16 (16.3%)	45 (45.9%)	13 (13.3%)	13 (13.3%)	44 (44.9%)		41 (41.8%)
Male controls	51 (38.9%)	66 (50.4%)		14 (10.7%)	13 (9.9%)	66 (50.4%)	52 (39.7%)	18 (13.7%)	68 (51.9%)		45 (34.4%)
AS											
Female patients	**7 (19.4%)^a^**	23 (63.9%)		6 (16.7%)	10 (27.8%)	16 (44.4%)	10 (27.8%)	**8 (22.2%)^b^**	22 (61.1%)		6 (16.7%)
Male patients	47 (44.3%)	48 (45.3%)		11 (10.4%)	11 (10.4%)	57 (53.8%)	38 (35.8%)	18 (17.0%)	46 (43.4%)		42 (39.6%)
Female controls	53 (54.1%)	34 (34.7%)		11 (11.2%)	16 (16.3%)	45 (45.9%)	37 (37.8%)	13 (13.3%)	44 (44.9%)		41 (41.8%)
Male controls	51 (38.9%)	66 (50.4%)		14 (10.7%)	13 (9.9%)	66 (50.4%)	52 (39.7%)	18 (13.7%)	68 (51.9%)		45 (34.4%)
PsA											
Female patients	26 (41.9%)	27 (43.5%)		9 (14.5%)	8 (12.9%)	29 (46.8%)	25 (40.3%)	10 (16.4%)	30 (49.2%)		21 (34.4%)
Male patients	29 (45.3%)	26 (40.6%)		9 (14.1%)	12 (18.8%)	27 (42.2%)	25 (39.1%)	11 (17.2%)	29 (45.3%)		24 (37.5%)
Female controls	53 (54.1%)	34 (34.7%)		11 (11.2%)	16 (16.3%)	45 (45.9%)	37 (37.8%)	13 (13.3%)	44 (44.9%)		41 (41.8%)
Male controls	51 (38.9%)	66 (50.4%)		14 (10.7%)	12 (18.8%)	66 (50.4%)	52 (39.7%)	18 (13.7%)	68 (51.9%)		45 (34.4%)

^a^CMH: p=0.001, p_c_=0.032, OR=0.47, CI_95%_=(0.30–0.74); groupwise (females): p_c_=0.002; ^b^CMH: p = 0.001, p_c_ = 0.027, OR = 0.46, CI_95%_ = (0.29–0.72); groupwise (females): p_c_=0.038. RA, rheumatoid arthritis; AS, ankylosing spondylitis; PsA, psoriatic arthritis; CMH, Cochran-Mantel-Haenszel χ^2^ test; p_c_, value with Bonferroni correction.

**Figure 1:**
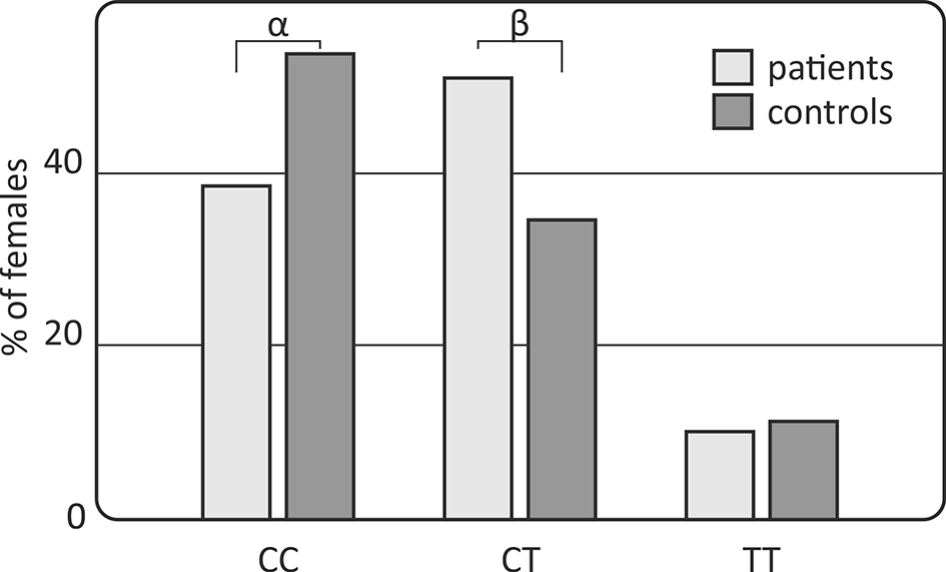
Association of the *IL-33* rs10975519 polymorphism with rheumatoid arthritis susceptibility in females. Differences are indicated as ^α^CC *vs* CT+TT, *p*=0.008, *p*_c_=NS, OR=0.54, CI_95%_=(0.33; 0.86); ^β^CT *vs* CC+TT, *p*=0.004, *p*_c_=NS, OR=1.97, CI_95%_=(1.22; 3.25); NS, non-significant; *p*_c_, value with Bonferroni correction.

However, gender-stratified analysis of the genotype and allele distributions revealed differences between female RA patients and female controls regarding the *IL-33* rs10975519 polymorphism (**Figure 1**). The *IL-33* rs10975519 CC genotype was less frequent among female patients than female controls (*p* = 0.008, *p*_c_ = NS, OR = 0.54, CI_95%_ = 0.33–0.86). On the contrary, female patients more frequently possessed the *IL-33* rs10975519 CG genotype than female controls (*p* = 0.004, *p*_c_ = NS, OR = 1.97, CI_95%_ = 1.22–3.25). No association remained significant after Bonferroni correction, however. No gender-dependent associations were detected between *IL-33* rs16924159 or rs7044343 and disease risk in the RA cohort.

### Gender-Stratified Analysis of the *IL-33* Genotype and Allele Frequencies in the RA, AS, and PsA Patients

A significant difference in the genotype and allele distribution was found for the *IL-33* rs10975519 genetic variant in the AS patients (**Table 2**). The frequency of the *IL-33* rs10975519 CC genotype was increased in male AS patients compared to females (*p*=0.001, *p*_c_=0.032, OR = 0.47, CI_95%_ = 0.30–0.74). The comparison between females and males diagnosed with AS in accordance with the *IL-33* rs7044343 polymorphism also revealed significant relationships. A frequency of the *IL-33* rs7044343 CC genotype was increased in the females with AS, as compared to the males (*p*=0.001, *p*_c_ = 0.027, OR = 0.46, CI_95%_ = 0.29–0.72). The analysis involving the *IL-33* rs16924159 SNP polymorphism in relation to the patients’ gender showed no significant associations.

The gender stratification analysis did not show any associations with any of the studied *IL-33* genetic variants in the RA and PsA patients (**Table 3**).

**Table 3:** Comparison of the *IL-33* genotypes and alleles frequencies between patients diagnosed with rheumatoid arthritis, ankylosing spondylitis, psoriatic arthritis, and healthy subjects.

	Controls	RA	AS	PsA
rs10975519				
C	308 (67.2%)	611 (65.5%)	179 (62.6%)	163 (64.7%)
T	150 (32.8%)	321 (34.4%)	54 (37.4%)	89 (35.3%)
CC	104 (45.4%)	186 (39.9%)	54 (37.8%)	55 (43.7%)
CT	100 (43.7%)	239 (51.3%)	71 (49.7%)	53 (42.1%)
TT	25 (10.9%)	41 (8.8%)	18 (12.6%)	18 (14.3%)
rs16924159				
A	169 (36.9%)	358 (38.4%)	116 (40.6%)	96 (38.1%)
G	289 (63.1%)	574 (61.6%)	170 (59.4%)	156 (61.9%)
AA	29 (12.7%)	65 (13.9%)	21 (14.7%)	20 (15.9%)
AG	111 (48.5%)	228 (48.9%)	74 (51.7%)	56 (44.4%)
GG	89 (38.9%)	173 (37.1%)	48 (33.6%)	50 (39.7%)
rs7044343				
C	174 (38.0%)	362 (38.8%)	122 (42.7%)	101 (40.4%)
T	284 (62.0%)	570 (61.2%)	164 (57.3%)	149 (59.6%)
CC	31 (13.5%)	55 (11.8%)	27 (18.9%)	21 (16.8%)
CT	112 (48.9%)	252 (54.1%)	68 (47.6%)	59 (47.2%)
TT	86 (37.6%)	159 (34.1%)	48 (33.6%)	45 (36.0%)

RA, rheumatoid arthritis; AS, ankylosing spondylitis; PsA, psoriatic arthritis.

### Effect of *IL-33* Polymorphisms on the Effectiveness of Anti-TNF Therapy in Patients Diagnosed With RA, AS, and PsA

The anti-TNF therapy clinical outcome was associated with the *IL-33* rs16924159 variant in the RA patients (**Table 4**). The *IL-33* rs16924159 AA genotype was more frequently observed among the RA patients with worse response to anti-TNF agents after 24 weeks than the other genotypes (*p* = 0.030, *p*_c_ = NS, OR = 1.97, CI_95%_ = 1.05–3.72). Moreover, the presence of the *IL-33* rs16924159 GG genotype was associated with a good response to anti-TNF treatment at the 24th week (*p* = 0.046, *p*_c_ = NS, OR = 1.98, CI_95%_ = 0.99–3.98). However, with Bonferroni adjustment no effect reached significance. There were no differences between the other two studied *IL-33* polymorphisms and the clinical outcomes of the anti-TNF treatment among the RA patients.

**Table 4:** Analysis of the *IL-33* genotypes distributions with regard to therapeutic response to anti-TNF agents in rheumatoid arthritis, ankylosing spondylitis and psoriatic arthritis patients.

		rs10975519			rs16924159			rs7044343	
	CC	CT	TT	AA	AG	GG	CC	CT	TT
RA									
12^th^ week									
Responders	26 (37.1%)	39 (55.7%)	5 (7.1%)	10 (14.3%)	33 (47.1%)	27 (38.6%)	7 (10.0%)	39 (55.7%)	24 (34.3%)
Non-responders	145 (40.7%)	181 (50.8%)	30 (8.4%)	46 (12.9%)	178 (50.0%)	132 (37.1%)	42 (11.8%)	193 (54.2%)	121 (34.0%)
24^th^ week									
Responders	98 (40.7%)	125 (51.9%)	18 (7.5%)	**25 (10.4%)^a^**	124 (51.5%)	**92 (38.2%)^b^**	27 (11.2%)	129 (53.5%)	85 (35.3%)
Non-responders	56 (38.6%)	76 (52.4%)	13 (9.0%)	27 (18.6%)	68 (46.9%)	50 (34.5%)	16 (11.0%)	81 (55.9%)	48 (33.1%)
AS									
12^th^ week									
Responders	34 (32.4%)	56 (53.3%)	15 (14.3%)	18 (17.1%)	50 (47.6%)	37 (35.2%)	23 (21.9%)	51 (48.6%)	31 (29.5%)
Non-responders	18 (52.9%)	15 (44.1%)	1 (2.9%)	3 (8.8%)	22 (64.7%)	9 (26.5%)	2 (5.9%)	17 (50.0%)	15 (44.1%)
24^th^ week									
Responders	46 (36.8%)	64 (51.2%)	15 (12.0%)	**15 (12.0%)^c^**	66 (52.8%)	**44 (35.2%)^d^**	24 (19.2%)	60 (48.0%)	41 (32.8%)
Non-responders	3 (37.5%)	4 (50.0%)	1 (12.5%)	4 (36.4%)	3 (27.3%)	4 (36.4%)	1 (12.5%)	5 (62.5%)	2 (25.0%)
PsA									
12^th^ week									
Responders	32 (41.6%)	35 (45.5%)	10 (13.0%)	13 (16.9%)	35 (45.5%)	29 (37.7%)	15 (19.5%)	37 (48.1%)	25 (32.5%)
Non-responders	12 (40.0%)	14 (46.7%)	4 (13.3%)	5 (16.7%)	14 (46.7%)	11 (36.7%)	6 (20.0%)	14 (46.7%)	10 (33.3%)
24^th^ week									
Responders	38 (47.5%)	33 (41.3%)	9 (11.3%)	13 (16.3%)	36 (45.0%)	31 (38.8%)	13 (16.5%)	37 (46.8%)	29 (36.7%)
Non-responders	12 (46.2%)	11 (42.3%)	3 (11.5%)	4 (15.4%)	12 (46.2%)	10 (38.5%)	4 (15.4%)	12 (46.2%)	10 (38.5%)

^a^AA vs AG+GG, p = 0.030, p_c_ = NS, OR = 0.51, CI_95%_ = 0.27–0.95; ^b^GG vs AA, p = 0.046, p_c_ = NS, OR = 1.98, CI_95%_=(0.99–3.98); ^c^AA vs AG+GG, p = 0.015, p_c_ = NS, OR = 0.14, CI_95%_ = (0.02–0.83); ^d^GG vs AA, p=0.024, p_c_=NS, OR=11.22, CI_95%_ = 1.01–588.34.

RA, rheumatoid arthritis; EULAR, European League Against Rheumatism; AS, ankylosing spondylitis; BASDAI, Bath Ankylosing Spondylitis Disease Activity Index; PsA, psoriatic arthritis; PsARC, Psoriatic Arthritis Response Criteria; NS, non-significant; p_c_, value with Bonferroni correction.

Differences were also detected between the *IL-33* rs16924159 polymorphism and anti-TNF therapy efficacy in the AS patients (**Table 4**). After 24 weeks of treatment, a lack of response was more frequently observed in patients carrying the AA genotype than the other genotypes (*p* = 0.015, *p*_c_ = NS, OR = 7.15, CI_95%_ = 1.20–42.87). Additionally, an elevated frequency of the *IL-33* rs16924159 A allele was detected among patients with no response to anti-TNF agents after 24 weeks (*p* = 0.032, *p*_c_ = NS, OR = 3.51, CI_95%_ = 1.08–13.31). On the contrary, *IL-33* rs16924159 GG genotype frequencies were increased in patients who responded to anti-TNF treatment after 24 weeks compared to the AA genotype (*p* = 0.024, *p*_c_ = NS, OR = 11.22, CI_95%_ = 1.01–588.34).

There were no differences between the other two studied *IL-33* polymorphisms and the anti-TNF therapy outcomes among the RA and AS patients. No significant relationships in the genotype or allele distribution of the studied *IL-33* polymorphism and anti-TNF agent efficacy were found for the PsA patients (**Table 4**).

### Clinical Parameters of the RA, AS, and PsA Patients and *IL-33* Genotype and Allele Distributions

**Figure 2:**
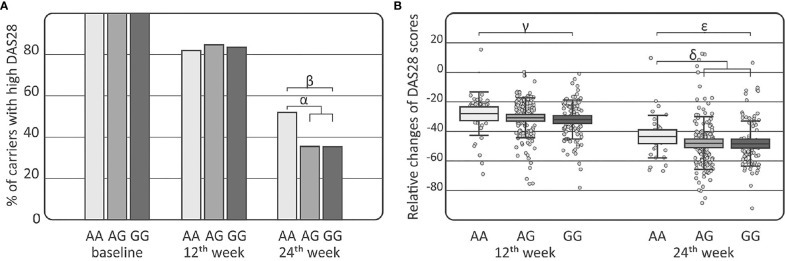
Association of the *IL-33* rs16924159 polymorphism with disease activity in rheumatoid arthritis patients. **(A)** Comparison of *IL-33* rs16924159 genotype frequencies with DAS28 score at baseline as well as at 12th and 24th weeks after commencing of anti-TNF therapy; **(B)** Comparison of *IL-33* rs16924159 genotype frequencies with relative change in DAS28 between baseline and 12th week, as well as 24th week after commencing of anti-TNF therapy; Differences are indicated as; ^α^AA *vs* AG+GG, *p*=0.023, *p*_c_=NS, OR= 1.97, CI_95%_=(1.06; 3.67); ^β^GG *vs* AA, *p*=0.049, *p*_c_=NS, OR=0.51, CI_95%_=(0.25; 1.00); ^γ^AA *vs* GG, *p*=0.036, *p*_c_=NS, W=2396.5; ^δ^AA *vs* AG+GG, *p*=0.030, *p*_c_=NS, W=5265.5; ^ϵ^GG vs AA, *p*=0.016, *p*_c_=NS, W=2301.0; DAS28, disease activity score 28; NS, non-significant; *p*_c_, value with Bonferroni correction.

Comparisons of the *IL-33* genotypes and alleles among the RA, AS, and PsA patients were performed using clinical laboratory parameters. Association between DAS28 and the distribution of the *IL-33* rs16924159 genotype was observed in the RA patients at week 24 of treatment (**Figure 2**). The *IL-33* rs16924159 AA genotype was more common among RA patients with higher DAS28 after 24 weeks of therapy than the other genotypes (*p* = 0.023, *p*_c_ = NS, OR = 1.97, CI_95%_ = 1.06–3.67). On the contrary, RA patients bearing the *IL-33* rs16924159 GG genotype had lower values of DAS28 at week 24 of anti-TNF treatment than patients carrying the other genotypes (*p* = 0.049, *p*_c_ = NS, OR = 1.97, CI_95%_ = 1.00–3.93). After Bonferroni correction, the associations were no longer significant.

Differences were detected in the RA patients between the *IL-33* rs16924159 genotype and relative changes in DAS28 values during treatment (**Figure 2**). Patients bearing the *IL-33* rs16924159 GG genotype had larger DAS28 reductions at week 12 of anti-TNF treatment than carriers of the AA genotype (*p* = 0.036, *p*_c_ = NS, W = 2396.5). This trend was also observed after 24 weeks of treatment. Lesser relative changes in DAS28 values were observed in patients with the *IL-33* rs16924159 AA genotype than the other genotypes (*p*=0.030, *p*_c_=NS, W=5265.5). Patients homozygous for the *IL-33* rs16924159 GG genotype demonstrated a greater decrease in DAS28 values between baseline and week 24 than patients possessing the AA genotype (*p* = 0.016, *p*_c_ = NS, W = 2301.0). No other studied *IL-33* polymorphism was associated with any clinical parameters in the gender-stratified or whole RA cohort.

A significant association in the *IL-33* rs16924159 genotype distribution with regard to disease activity was found in the AS patients (**Table 5**). AS patients carrying the *IL-33* rs16924159 AA genotype displayed higher baseline BASDAI scores than patients possessing the other genotypes (*p* = 0.001, *p*_c_ = 0.027, W = 1821.0). On the contrary, lower baseline BASDAI scores were observed in AS patients bearing the *IL-33* rs16924159 GG genotype than AA homozygous patients (*p* = 0.003, *p*_c_ = 0.081, W = 718.0).

**Table 5:** Analysis of the *IL-33* genotypes distributions with regard to baseline clinical parameters of rheumatoid arthritis, ankylosing spondylitis and psoriatic arthritis patients.

		rs10975519			rs16924159			rs7044343	
	CC	CT	TT	AA	AG	GG	CC	CT	TT
RA									
RF+	85 (65.4%)	126 (71.2%)	20 (64.5%)	33 (70.2%)	107 (65.2%)	91 (71.7%)	25 (62.5%)	136 (73.1%)	70 (62.5%)
anti-CCP+	104 (89.7%)	148 (89.7%)	28 (93.3%)	43 (93.5%)	139 (91.4%)	98 (86.7%)	36 (94.7%)	156 (90.2%)	88 (88.0%)
CRP	24.4 (± 34.5)	22.1 (± 30.5)	26.7 (± 47.5)	22.7 (± 27.5)	22.6 (± 33.4)	24.8 (± 36.8)	25.5 (± 43.5)	23.0 (± 31.0)	23.4 (± 34.9)
VAS	79.4 (± 10.3)	76.1 (± 12.5)	77.6 (± 13.4)	74.8 (± 12.3)	77.4 (± 12.1)	78.8 (± 11.0)	76.4 (± 12.9)	77.0 (± 12.6)	78.8 (± 10.0)
DAS28	6.4 (± 0.7)	6.4 (± 0.7)	6.5 (± 0.7)	6.4 (± 0.7)	6.4 (± 0.7)	6.5 (± 0.7)	6.4 (± 0.7)	6.4 (± 0.7)	6.4 (± 0.6)
AS									
CRP	27.6 (± 28.5)	34.8 (± 77.0)	22.4 (± 28.7)	20.3 (± 17.7)	33.5 (± 75.2)	30.5 (± 28.3)	23.5 (± 27.6)	32.6 (± 75.1)	29.9 (± 29.8)
VAS	80.7 (± 9.8)	81.0 (± 11.5)	78.5 (± 10.9)	84.1 (± 7.1)	80.3 (± 10.3)	79.5 (± 11.1)	80.2 (± 10.6)	80.6 (± 10.0)	80.8 (± 11.4)
BASDAI	7.6 (± 1.4)	7.8 (± 1.3)	7.2 (± 1.2)	**8.2 (± 1.2)^a^**	7.5 (± 1.4)	**7.5 (± 1.4)^b^**	7.5 (± 1.1)	7.7 (± 1.2)	7.7 (± 1.3)
PsA									
CRP	12.0 (± 17.6)	13.1 (± 17.1)	10.4 (± 16.2)	10.2 (± 12.5)	11.4 (± 17.3)	14.0 (± 18.6)	9.9 (± 15.2)	14.1 (± 17.7)	11.1 (± 17.2)
VAS	72.6 (± 14.8)	72.9 (± 15.9)	68.9 (± 19.0)	65.5 (± 18.6)	72.2 (± 14.4)	75.1 (± 15.6)	71.2 (± 18.3)	72.6 (± 15.4)	71.8 (± 15.4)
BASDAI	7.2 (± 1.4)	7.7 (± 1.4)	7.2 (± 1.2)	8.4 (± 1.1)	7.3 (± 1.4)	7.3 (± 1.4)	7.5 (± 1.1)	7.6 (± 1.6)	7.3 (± 1.3)

^a^AA vs AG+GG, p=0.001, p_c_=0.027, W=1821.0; ^b^GG vs AA, p = 0.003, p_c_ = NS, W = 718.0.

RA, rheumatoid arthritis; SJC, swollen joints count; TJC, tender joints count; RF, rheumatoid factor; anti-CCP, anti cyclic citrullinated peptide antibody; CRP, C-reactive protein; VAS, visual analog scale; DAS28, disease activity score 28; AS, ankylosing spondylitis; BASDAI, Bath Ankylosing Spondylitis Disease Activity Index; PsA, psoriatic arthritis; NS, non-significant; p_c_, value with Bonferroni correction.

A comparison of clinical parameters with genotype frequencies and alleles of the *IL-33* variants did not show any associations in the PsA patients (**Table 5**).

## Discussion

A growing body of evidence highlights the role of the IL-33 signaling pathway in inflammatory arthritis. Significantly increased IL-33 levels have been detected in RA patients’ serum and synovial fluid, and IL-33 levels have been shown to positively correlate with disease severity, rheumatoid factor, and anticitrullinated protein antibodies (37–39). Furthermore, a reduction in serum IL-33 concentration in RA patients after anti-TNF therapy has been reported (38, 40). In addition, decreased levels of IL-33 have not been observed in patients unresponsive to TNF inhibitors (37). It has also been reported that TNF stimulates the IL-33 expression on both mRNA and protein levels in cultured synovial fibroblasts derived from RA patients (41–43). On the contrary, it has been shown that IL-33 affects TNF-dependent effects, enhancing the production of pro-inflammatory mediators, including IL-6, IL-8, and monocyte chemotactic protein-1 (MCP-1) and the pro-destructive molecules matrix metalloproteinase-1 (MMP−1), MMP-3, and TIMP-1 (41).

In the present study, the *IL-33* rs16924159 polymorphism was associated with anti-TNF therapy efficacy and clinical parameters in RA and AS patients. The *IL-33* rs16924159 AA genotype was correlated with higher disease activity and worse clinical outcomes in RA patients treated with TNF inhibitors. AS patients carrying the *IL-33* rs16924159 AA genotype had higher disease activity and a worse response to anti-TNF therapy; these results indicate a deleterious role of the *IL-33* rs16924159 AA genotype in RA and AS. A significant association was also found for the *IL-33* rs10975519 genetic variant. The *IL-33* rs10975519 CC genotype was associated with a decreased risk of developing RA among females. We did not find in the literature any gene candidate study investigating these SNPs in RA or PsA patients. One other study by Fan et al. addressed the relationships between the *IL-33* rs16924159 and rs10975519 polymorphisms and AS development; however, all subjects in this study were of Chinese ethnicity (44). The study reported a correlation between the rs10975519 CC genotype and a diminished predisposition to AS, but no association between *IL-33* rs16924159 and AS susceptibility was found (44). The *IL-33* rs10975519 C allele was previously associated with higher susceptibility to ischemic stroke in a Chinese cohort by Guo et al. (45). The *IL-33* rs10975519 variant was also studied in Chinese patients diagnosed with autoimmune thyroid diseases, systemic lupus, and coronary artery disease, although no significant relationships were revealed (46–48). The *IL-33* rs16924159 AA polymorphism was found to be associated with an increased risk of recurrent miscarriage (49). On the other hand, the rs16924159 A allele has been identified as a protective allele in asthma development (50). No association has been found between the *IL-33* rs16924159 and coronary artery disease or coronary heart disease in Mexican or Chinese populations, respectively (51, 52). There is also no significant relationship between this polymorphism and the risk of ischemic stroke (45). *IL-33* loci did not reach genome-wide level of statistical significance in previous GWAS studies in RA patients (53). No associations were also found with respect to disease susceptibility in previous GWAS studies involving AS and PsA patients (54–56). These results are in line with the results from the present study. None of the studied *IL-33* genetic variants was associated with RA, AS, or PsA risk in the whole cohort of patients. However, in the present study, significant association was observed between the *IL-33* rs10975519 genetic variant and predisposition to RA in females. Alas, in the aforementioned GWAS studies sex-stratified analyses were not applied, so no conclusions can be drawn. In the present study, significant relationships were also observed between the *IL-33* genetic variants and anti-TNF efficacy in RA as well as AS patients. However, GWAS studies investigating outcome of anti-TNF therapy in RA patients didn’t identify genome-wide significant association for *IL-33* loci (57, 58). Interestingly, in a study investigating transcriptomic profile, *IL-33* genome-level expression was found significantly upregulated in RA patients that responded to anti-TNF therapy (59). The inconsistency between the studies might be attributed to differences in inclusion and outcome criteria between studies or false-positive associations obtained. Also, anti-TNF response is considered to be polygenic with many small-effect variants, which might be missed in GWAS analysis. Therefore, results derived from this study require validation in larger patient cohorts from Caucasian population.

Herein, no association was found between the *IL-33* rs7044343 polymorphism and RA susceptibility or anti-TNF treatment efficacy. Interestingly, a previous study using a Chinese cohort reported a significant association of the *IL-33* rs7044343 CC genotype with a decreased predisposition to RA (60). The *IL-*33 rs7044343 C allele has also been shown to be strongly associated with a diminished risk of Alzheimer’s disease in a study encompassing various populations (61). A meta-analysis performed by Zhong et al. also noted the *IL-33* rs7044343 C allele as a protective factor for Alzheimer disease (AD) development (62). However, no significant association was found in another study regarding Chinese patients diagnosed with AD (63). On the contrary, the rs7044343 C allele was identified as a risk factor for susceptibility to Behçet’s disease and systemic sclerosis (64, 65). In line with these findings, the *IL-33* rs7044343 T allele has been associated with a decreased risk of coronary artery disease (51). The *IL-33* rs7044343 genetic variant was also examined for associations with asthma development in a Tunisian cohort; however, no significant relationships were found (66). Although, several other polymorphisms within the *IL-33* gene have been shown to be significantly associated with asthma susceptibility in various populations (50). A significant relationship was also found between the *IL-33* rs7044343 polymorphism and ischemic stroke in a Chinese cohort (67). On the contrary, there were no associations between the *IL-33* rs7044343 polymorphism and inflammatory bowel disease or idiopathic achalasia in an Italian cohort (68, 69). The *IL-33* rs7044343 polymorphism was also investigated in patients diagnosed with giant cell arteritis and chronic obstructive pulmonary disease; however, no significant correlations were detected (70, 71).

The discord between our studies’ results and the aforementioned studies using Chinese cohorts most likely comes from genetic differences between the studied populations or inadequate sample sizes, particularly for AS and PsA cohorts. Indeed, the present study’s main limitation was the limited sample size, possibly resulting in insufficient power for detecting associations.

Among the AS patient cohort, gender-specific associations in genotype distributions were found for all two studied genetic variants of *IL-33*. The *IL-33* rs1097559 CC genotype was less frequently observed among female AS patients than males, and the frequency of the *IL-33* rs7044343 TT genotype was significantly decreased in female patients. AS affects men more frequently than women, with male to female ratio of 3:1 (72, 73). Gender-related differences have been also detected with regard to clinical manifestation, radiographic damage and efficacy of biologic treatment. Female patients have higher disease activity defined as BASDAI and worse response to biologic treatment as compared to males (73–76). On the other hand, men are more likely to experience severe radiographic damage (77, 78). However, molecular mechanisms underlying these differences are currently unknown. No GWAS study performed to date on AS patients included sex-specific analyses. Some gene candidate studies reported gender-specific associations between studied genetic variants and AS. Polymorphisms within *ANKH* gene were found to be associated with AS susceptibility in a gender-dependent manner (79). Also, sex-related differences were observed in a haplotype distribution of specific tissue non-specific alkaline phosphatase (*TNAP*) gene (80). In addition, in the study conducted by Gracey et al., authors observed that gene expression profiles in AS patients differed between males and females (81).

The current study constitutes the first report of associations between *IL-33* gene polymorphisms and rheumatic diseases in a Caucasian population. The results indicated that *IL-33* polymorphisms might be associated with rheumatic disease risk and anti-TNF treatment outcomes in Caucasians. Nevertheless, additional studies with larger sample sizes and different populations are needed to validate these findings and to establish the exact role of *IL-33* polymorphisms in rheumatic diseases.

## Data Availability Statement

The data sets presented in this study can be found in online repositories. The names of the repository/repositories and accession number(s) can be found below: https://cloud.hirszfeld.pl/index.php/s/tX7JWbq5CPZxHJi, tX7JWbq5CPZxHJi.

## Ethics Statement

The studies involving human participants were reviewed and approved by the Wroclaw Medical University Ethics Committee. The patients/participants provided their written informed consent to participate in this study.

## Author Contributions

MI contributed to the conception and design of the study, genotyping, and statistical analyses and drafted and finalized the manuscript. JW performed most of the genotyping of patient and control samples, participated in the collection of literature data. JS, KK, RS, BB, and SJ provided clinical data and patient samples. MC-M prepared the control samples and their description. KB-K contributed to the conception and design of the study, drafted and finalized the manuscript, and secured funding All authors contributed to the article and approved the submitted version.

## Funding

This work was supported by a grant from the National Science Centre, Poland (grant number 2016/21/B/NZ5/01901).

## Conflict of Interest

The authors declare that the research was conducted in the absence of any commercial or financial relationships that could be construed as a potential conflict of interest.
